# The Effect of Fiber Bleaching Treatment on the Properties of Poly(lactic acid)/Oil Palm Empty Fruit Bunch Fiber Composites

**DOI:** 10.3390/ijms150814728

**Published:** 2014-08-22

**Authors:** Marwah Rayung, Nor Azowa Ibrahim, Norhazlin Zainuddin, Wan Zuhainis Saad, Nur Inani Abdul Razak, Buong Woei Chieng

**Affiliations:** 1 Department of Chemistry, Faculty of Science, Universiti Putra Malaysia, Serdang 43400, Malaysia; E-Mails: norhazlin@upm.edu.my (N.Z.); niar_02094@yahoo.com (N.I.A.R.); chieng891@gmail.com (B.W.C.); 2 Department of Microbiology, Faculty of Biotechnology and Biomolecular Sciences, Universiti Putra Malaysia, Serdang 43400, Malaysia; E-Mail: zuhainis@upm.edu.my

**Keywords:** composite, PLA, OPEFB, bleaching treatment, hydrogen peroxide, fiber/matrix interfacial adhesion, colorant

## Abstract

In this work, biodegradable composites from poly(lactic acid) (PLA) and oil palm empty fruit bunch (OPEFB) fiber were prepared by melt blending method. Prior to mixing, the fiber was modified through bleaching treatment using hydrogen peroxide. Bleached fiber composite showed an improvement in mechanical properties as compared to untreated fiber composite due to the enhanced fiber/matrix interfacial adhesion. Interestingly, fiber bleaching treatment also improved the physical appearance of the composite. The study was extended by blending the composites with commercially available masterbatch colorant.

## 1. Introduction

The majority of commodity plastics used today are made of non-biodegradable petroleum-based polymer [1]. The persistence of these materials in the environment beyond their functional life has resulted in a broad range of pollution, litter and waste disposal problems. Concern about the growing environmental issues and preservation of natural resources has stimulated the interest in biodegradable polymers based on renewable resources. Bio-based polymers offer environmental-friendly benefits as they have the capability to degrade naturally into organic substances without releasing any toxic components [2]. There are numbers of bio-based polymers that have been introduced commercially and available in the market. Among all, poly(lactic acid) (PLA) attracts enormous attention because of its versatility, biocompatibility and biodegradability characteristics.

PLA is a type of linear aliphatic thermoplastic polyester derived from renewable agricultural raw materials and commonly synthesized by two methods; ring opening polymerization of lactide and direct polycondensation of lactic acid [3]. PLA has good stiffness, strength and transparency and shows comparable properties in comparison with other petroleum based polymers such as polyethylene (PE), polystyrene (PS) and polypropylene (PP). The unique characteristics of PLA which can be processed similarly to polyolefins [4] make it an attractive option as an alternative to conventional polymers. Despite the excellent properties of PLA, relatively high production costs somewhat restrict its potential widespread application. One way to tackle this problem is through the incorporation of low cost, renewable and fully degradable natural filler such as oil palm empty fruit bunch (OPEFB) fiber to produce a cost effective composite.

In recent years, oil palm empty fruit bunch (OPEFB) fiber has received significant attention in the field of composite materials. OPEFB fiber is a waste product generated from the oil palm industry, available abundantly in Malaysia and its surrounding South East Asia countries [5]. In particular, OPEFB fiber attracts interest because of its environmental friendly nature, abundance, low density and most importantly low cost [6]. The incorporation of OPEFB fiber into PLA will optimize the use of oil palm and turn it from waste to value added product. Consequently, exploiting this waste will help to solve waste management problems, preserve natural resources and maintain ecological balance.

Even if PLA/OPEFB fiber composites are fully degradable, lightweight and cheaper, this combination presents several limitations. For instance, the incompatible character between polar-hydrophilic OPEFB and non-polar hydrophobic PLA matrix results in a poor fiber/matrix interfacial adhesion [7]. In addition, a typical mixing of native natural fiber/polymer matrix produces a dark brown color product, which limits its applications because the range of colors that can be obtained is limited. Therefore, considerable efforts have been made to enhance the composites performance.

A number of different techniques can be used to optimize the interfacial properties of a composite, such as surface modification of fiber via physical or chemical route and the use of an appropriate additive [8]. This study will demonstrate the efficiency of hydrogen peroxide (H_2_O_2_) as a bleaching agent. H_2_O_2_ has the capability to decolorize the fiber by removing lignin, hemicellulose and surface impurities. To date, there are limited numbers of studies that have been reported about bleached fiber composites. Thus, it is interesting to investigate whether or not fiber bleaching treatment can improve the properties of the composites in terms of appearance and performance. The aim of this work is to study the influence of fiber bleaching treatment with hydrogen peroxide on the properties of PLA/OPEFB composite. In addition, the use of masterbatch colorant to enhance the physical appearance of the composites is investigated.

## 2. Results and Discussion

### 2.1. Effect of Fiber Bleaching

**Figure 1:**
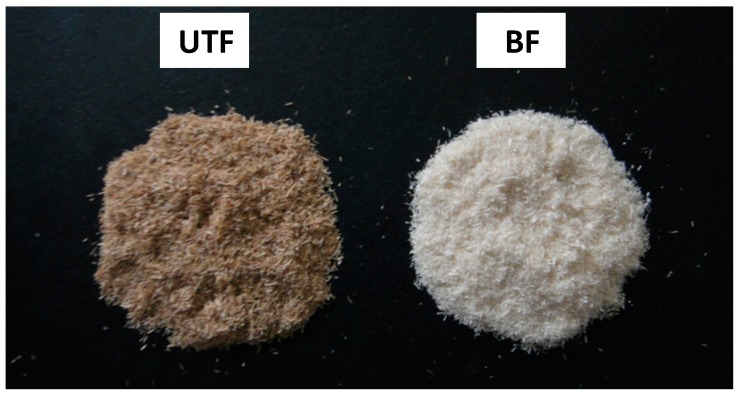
Optical images of untreated fiber (UTF) and bleached fiber (BF).

Bleaching treatment with hydrogen peroxide (H_2_O_2_) has a very pronounced effect on the brightness of the fiber as shown in Figure 1. The use of H_2_O_2_ as an oxidizing bleaching agent causes discoloration of fiber. Theoretically, perhydroxyl ions (HOO^−^) are generated by the dissociation of hydrogen peroxide in alkaline media and are responsible for the decolorization of the fiber (Equation (1)). These ions attack the light absorbing chromophoric groups of lignin and cellulose (carbonyl and conjugated carbonyl groups and quinones) [9]. The following equation shows the formation of perhydroxyl ions from the dissociation of hydrogen peroxide in alkaline medium [10].

H_2_O_2_ + OH^−^ → H_2_O + HOO^−^ (1)

### 2.2. Scanning Electron Microscopy (SEM) Analysis of Oil Palm Empty Fruit Bunch (OPEFB) Fiber

**Figure 2:**
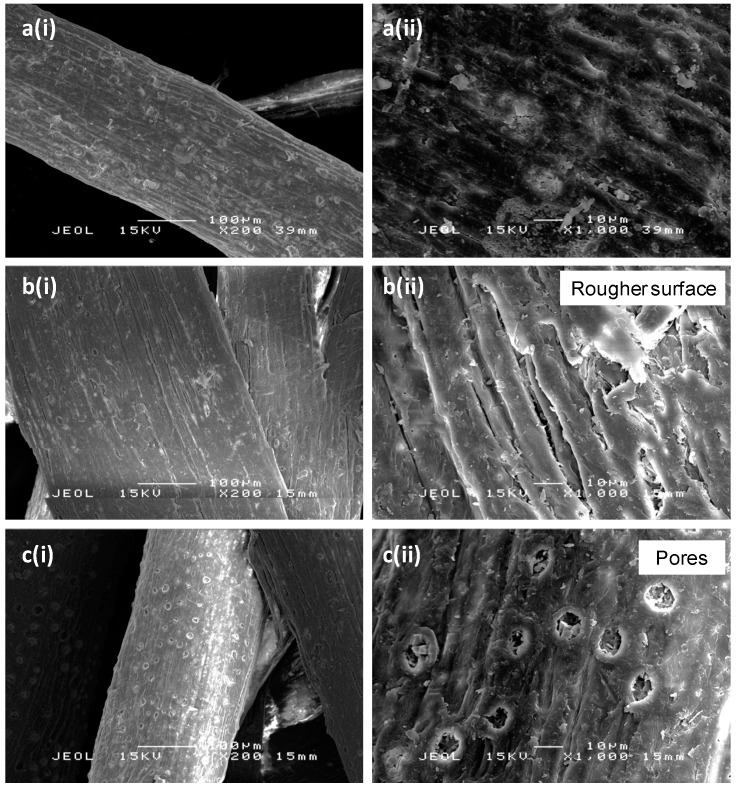
Scanning electron microscopy (SEM) micrographs of (**a**) UTF; (**b**) BF1; (**c**) BF2 at (**i**) 200× and (**ii**) 1000× magnification. BF1 and BF2 are the same sample but observed at different locations.

The scanning electron microscopy (SEM) micrographs for untreated and bleached oil palm empty fruit bunch (OPEFB) fiber can be seen in Figure 2. Some physical changes such as a rougher fiber surface can be observed (Figure 2b) after the fiber undergoes the bleaching treatment process. Changes in the fiber surface occur due to the removal of surface impurities, lignin and hemicellulose. Another observation revealed by the SEM micrographs of bleached fiber is the existence of pores on the fiber surface as presented in Figure 2c. The micrographs show that bleaching of fiber with hydrogen peroxide is capable of removing surface impurities and create rougher fiber surface, which is important to increase interfacial adhesion by mechanical interlocking [11].

### 2.3. Fourier Transform Infrared (FTIR) Analysis of OPEFB Fiber

**Figure 3:**
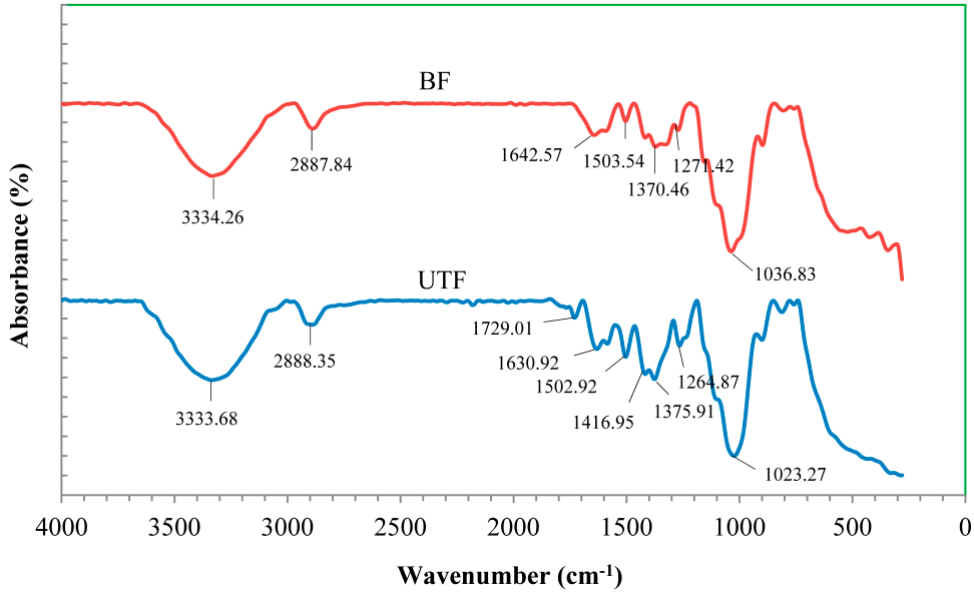
Fourier transform infrared (FTIR) spectra for untreated fiber (UTF) and bleached fiber (BF).

Fourier transform infrared (FTIR) spectroscopy is employed to study the functional groups of both untreated and bleached fiber and to monitor the effect of fiber treatment on structure and chemical changes of the lignocellulosic fiber. Figure 3 shows the IR spectra for untreated and bleached fiber. A broad peak can be observed at region 3600–3200 cm^−1^ indicating the presence of hydroxyl (O–H) group in both fibers. The absorbance peaks around 2900–2800 cm^−1^ were attributed to stretching of the C–H group. A peak at 1729.01 cm^−1^ was present for untreated fiber but not for bleached fiber. The peak corresponds to the C=O stretching of the acetyl group in hemicellulose [12]. The peak also indicates the ester linkage of the carboxylic group in the ferulic and *p*-coumeric acid of lignin and/or hemicellulose [13]. The lack of this peak for bleached fiber confirmed the removal of lignin and hemicellulose during the bleaching process. The intensity of the peak at 1502.92 cm^−1^ for untreated fiber decreased after the bleaching process. A peak at 1264.87 cm^−1^ for untreated fiber was shifted to 1271.24 cm^−1^ after the bleaching process and its intensity decreased. According to Joonobi *et al.* [13], that peak is attributed to the aryl group in lignin, thus the changes observed are due to the removal of lignin after fiber treatment. Finally, the intense peaks around 1070–1010 cm^−1^ region were due to the stretching of C–O and O–H groups [14].

### 2.4. X-ray Diffraction (XRD) Analysis of OPEFB Fiber

**Figure 4:**
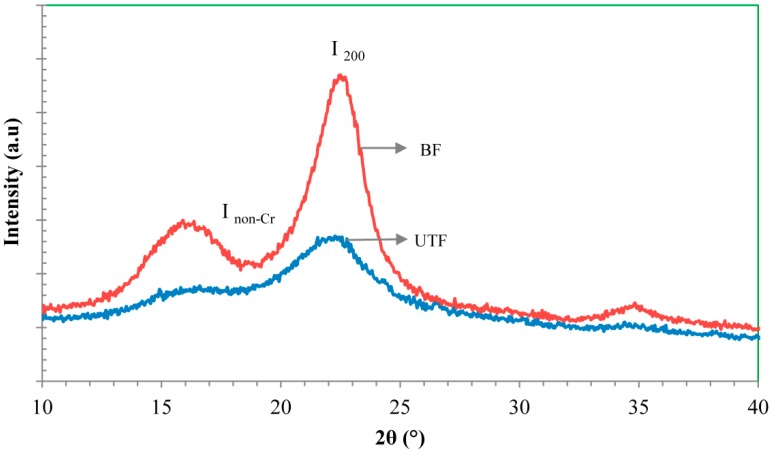
XRD patterns corresponding to untreated fiber (UTF) and bleached fiber (BF).

XRD analysis was carried out to examine the effect of bleaching treatment on the crystallinity of the fiber. The XRD diffractogram of untreated and bleached fibers are presented in Figure 4, where significant changes can be observed regarding the intensity of the peak for both types of fiber. A major diffraction peak at 2θ ranging between 22° and 23° can be seen, which corresponds to the crystallographic planes of cellulose I (I_200_). The peak near 2θ of 16° is the superposition of two peaks from the crystalline phase of the cellulose. The valley between the two peaks, (region around 2θ = 18°) represents the non-crystalline region of the fiber. From the diffractogram, the crystallinity index of fiber is determined using the empirical Segal equation as stated in the methodology section. The crystallinity index for the untreated fiber was found to be 37% and it increased to 61% for the bleached fiber. The increase in crystallinity is expected due to the removal of lignin and hemicellulose, the amorphous part in the fiber structure. A higher fiber crystallinity should lead to higher fiber tensile strength, which should improve the mechanical properties of the corresponding composite [15].

### 2.5. Biocomposites Preparation

**Figure 5:**
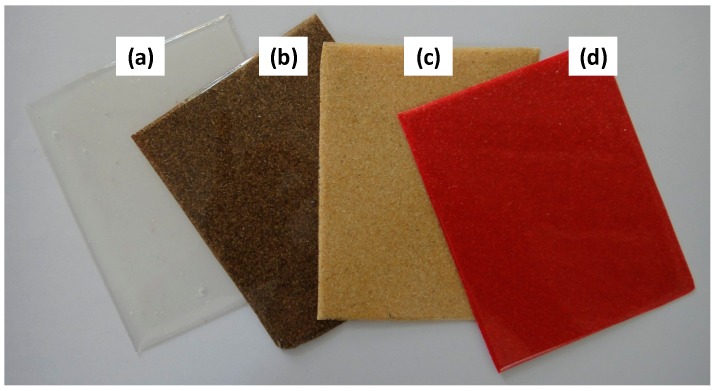
Image of (**a**) neat poly(lactic acid) (PLA); (**b**) PLA/UTF30; (**c**) PLA/BF30 and (**d**) PLA/masterbatch (MB)/BF30 composites.

PLA/OPEFB biocomposites from untreated and bleached fiber were successfully prepared by melt blending technique followed by compression molding. All the produced composites showed smooth surfaces indicating a uniform distribution of the fiber. Figure 5a shows the typical transparency of neat PLA. Upon the addition of untreated fiber, a brown sheet of composite was produced (Figure 5b, PLA/UTF). On the other hand, the combination of PLA with bleached fiber successfully produced a brighter color for the composite as shown in Figure 5c (PLA/BF). The change in color is believed due to the removal of lignin and hemicellulose during bleaching process. From this point of view, the bleaching of the fiber becomes a crucial step in order to produce composites with a better appearance that can be further processed to obtain various colors with the addition of masterbatch (MB) colorant (Figure 5d, PLA/MB/BF).

### 2.6. Effect of Fiber Bleaching on the Tensile Properties of Poly(lactic acid) (PLA)/OPEFB Fiber Biocomposites

**Figure 6:**
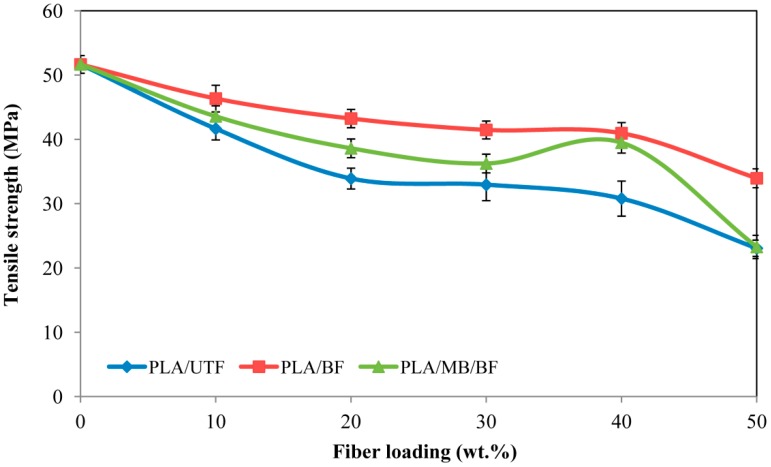
Tensile strength of PLA/UTF, PLA/BF and PLA/MB/BF composites.

The tensile strength of neat PLA, PLA/UTF, PLA/BF and PLA/MB/BF composites is reported in Figure 6. A decrease in tensile strength resulting from an increase in fiber loading was noticed for the three composites compared to PLA. This phenomenon could be attributed to a weak interfacial interaction between PLA and OPEFB fiber, which resulted into an inefficient stress transfer when stress is applied on a tensile specimen. Besides, the OPEFB used in short fiber form might not be able to support stress transferred from PLA, thus leading to strength reduction as fiber loading increases [16]. However, it is evident from the results that PLA/bleached fiber composites show significant improvement in tensile strength compared to the PLA/untreated fiber composites. According to Senawi *et al.* [17] treatment of fiber improves biocomposites properties by increasing wettability and interfacial bond strength with the matrix, explaining the enhancement in strength. On the other hand, it was observed from the tensile strength result that the addition of 0.5 wt. % masterbatch colorant resulted in a reduction of the overall composite strength. A similar observation was found by Byrne *et al.* [18].

**Figure 7:**
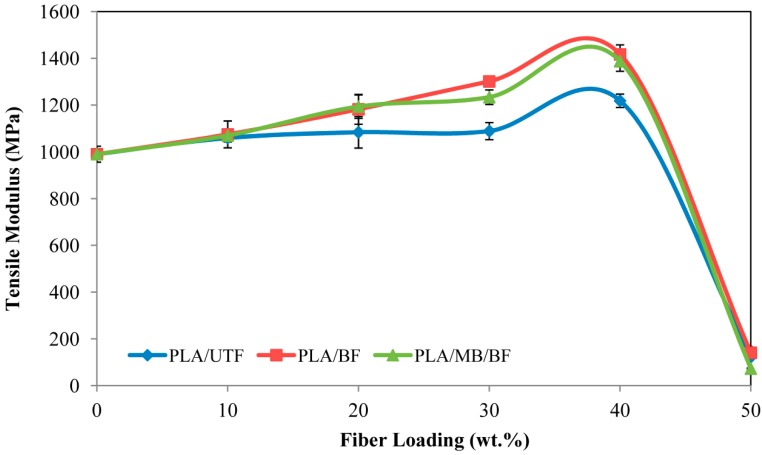
Tensile modulus of PLA/UTF, PLA/BF and PLA/MB/BF biocomposites.

Figure 7 illustrates the tensile modulus for neat PLA and the three composites. The modulus increased gradually as fiber loading was increased as well, for all fiber/matrix systems and reached a maximum at 40 wt. % fiber loading; it was higher than the modulus of PLA. A sudden drop of the tensile modulus was recorded at 50 wt. % fiber loading. That might have come from an insufficient wetting of fiber by the matrix. The increased in tensile modulus might be due to the fact that the fibers have a higher stiffness than the matrix. Based on the result, PLA/BF and PLA/MB/BF composites have higher tensile modulus compared to the untreated fiber composites. Indeed, the fiber treatment process removes lignin and hemicellulose, therefore increases the effectiveness of cellulose fiber as reinforcement [19]. Rosa *et al.* [20] reported the same pattern on tensile modulus with fiber treated in alkaline medium.

**Figure 8:**
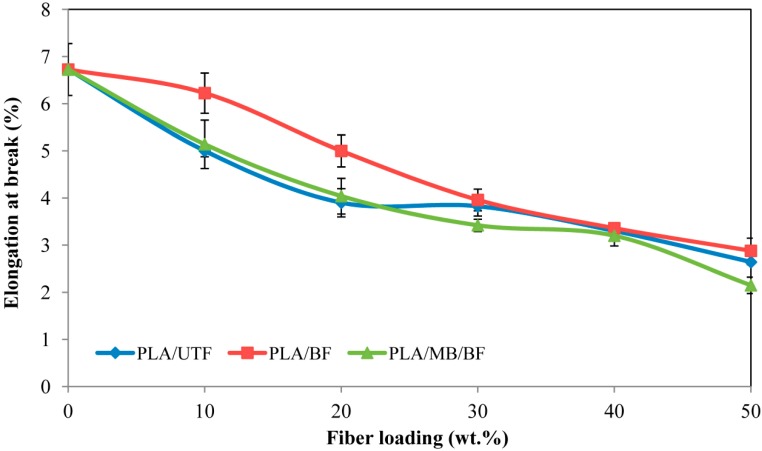
Elongation at break of PLA/UTF, PLA/BF and PLA/MB/BF biocomposites.

Figure 8 illustrates the elongation at break of neat PLA, PLA/UTF, PLA/BF and PLA/MB/BF composites. Generally, neat PLA has a higher elongation value compare to the composites. With the increment in fiber content from 0 to 50 wt. %, a gradual decrease in the elongation at break was observed due to the increasing of brittleness and stiffness of the composites, thus restricting the polymer chain mobility. The increment in strength and stiffness is usually accompanied by decrement in strain value. This finding is also in agreement with Chee *et al.* [21] who suggested that the fiber-matrix interaction is expected to diminish at higher fiber loading and being replaced by fiber-fiber interaction. Nevertheless, a notable improvement in elongation at break can be observed for bleached fiber composites compare to untreated fiber composites.

### 2.7. Effect of Fiber Bleaching on the Impact Properties of Biocomposites

**Figure 9:**
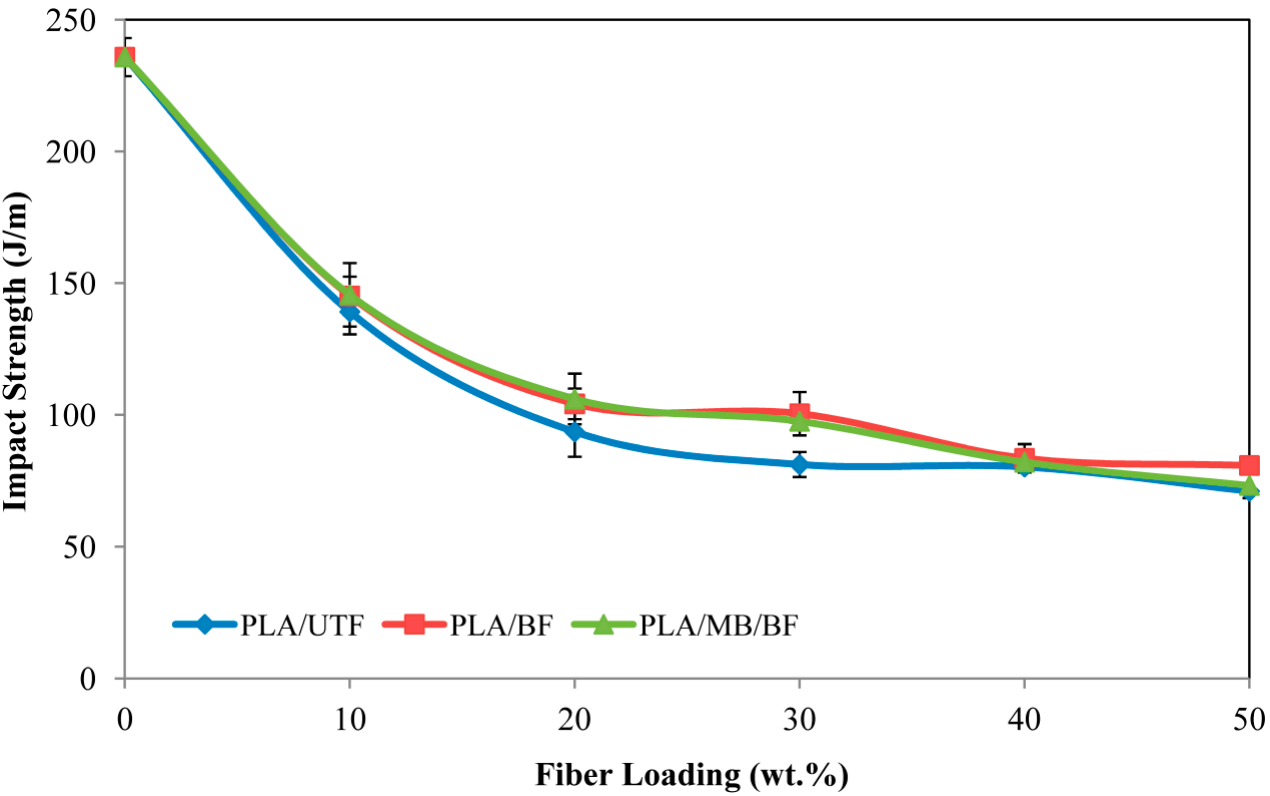
Impact strength of PLA/UTF, PLA/BF and PLA/MB/BF biocomposites.

The impact strength of un-notched neat PLA, PLA/UTF, PLA/BF and PLA/MB/BF as a function of fiber loading can be found in Figure 9. Lower impact strength compared to neat PLA and a gradual decrease is spotted with an increase of fiber content. According to Bengtsson *et al.* [22] the stiffer fiber will act as stress concentrators in the polymer matrix and reduce the crack initiation energy. Consequently, it will reduce the impact strength of the composites. Besides, with an increasing fiber content, the probability of fibers to agglomerate also increases, thus creating regions of stress concentration that require less energy to extend the fracture propagation [23].The presence of fiber will also reduce the energy absorbed by the composites during fracture propagation. The results also demonstrate an improvement in impact strength for bleached fiber composites compared to the untreated fiber composite. The improvement is likely due to the enhancement of fiber-matrix interaction which provides higher resistance to crack propagation [17].

### 2.8. SEM Analysis of Biocomposites

**Figure 10:**
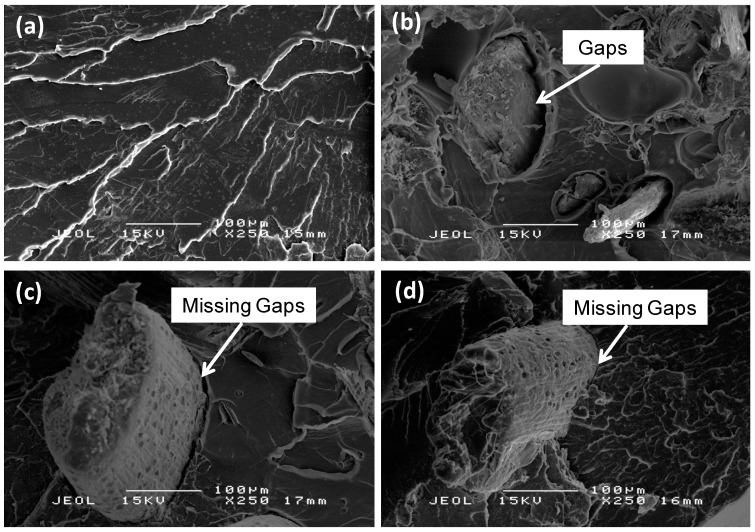
SEM micrographs of (**a**) PLA; (**b**) PLA/UTF; (**c**) PLA/BF; and (**d**) PLA/MB/BF.

The fracture surface of composites was studied to identify the nature of the failure mechanism and particularly to examine the level of fiber-matrix interaction [24]. Figure 10a shows the fracture surface micrograph of a tensile specimen of neat PLA. Meanwhile, SEM images of the tensile fracture surfaces of PLA/UTF, PLA/BF and PLA/MB/BF composites at 30 wt. % fiber loading are displayed in Figure 10b–d respectively. The micrographs corresponding to all three biocomposites show a visible fiber pull-out. The micrograph of untreated fiber composites (PLA/UTF) shows notable gaps between fiber and matrix. The gaps could be generated because of a debonding during mechanical test or because of a poor contact during composites preparation [25]. Either way, the gaps are an indication of poor fiber/matrix adhesion. In contradiction, the gaps are much less pronounced for bleached fiber composite (PLA/BF and PLA/MB/BF). This clearly shows the bleaching of fibers improves adhesion between the fibers and the matrix due to the removal of impurities and the generation of a rougher fiber surface after treatment process. Huda *et al.* [26] have made a similar conclusion and suggest that changes of surface topography could affect the fiber/matrix interfacial adhesion. A good fiber/matrix adhesion is required for effective stress transfer from the matrix to the fiber [19]. The enhancement in fiber/matrix adhesion explains the improvement in mechanical properties of bleached fiber composites compared to the untreated fiber composites.

### 2.9. Thermogravimetric Analysis (TGA) of Biocomposites

**Figure 11:**
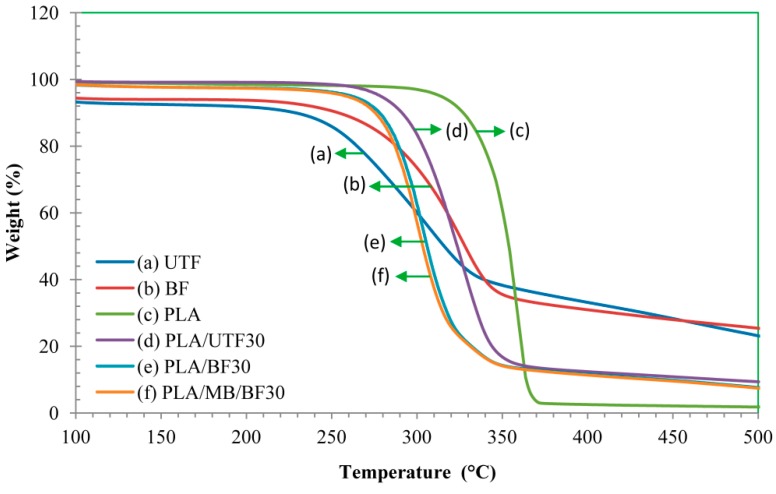
Thermogravimetric analysis (TGA) thermograms of neat PLA and biocomposites (70:30).

**Figure 12:**
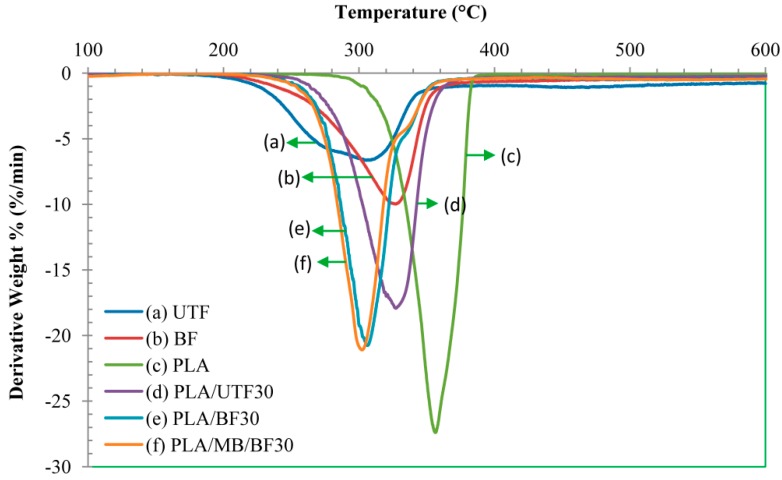
Derivative thermogravimetric (DTG) thermograms of neat PLA and biocomposites (70:30).

TGA was used in this study to investigate the thermal behavior of neat PLA and the composites. Figure 11 and Figure 12 show the thermogravimetric (TG) and derivative thermogravimetric (DTG) thermograms of neat PLA and the biocomposites at 30 wt. % fiber loading. The thermal degradation of neat PLA occurs in a single step degradation process around 270 °C and finishes at 390 °C. The decomposition of the composites happens at lower temperature. This is most likely attributed to the fact that the fiber has a lower decomposition temperature that possibly enhances the deformation of the crystalline structure of PLA, thus resulting in a reduction in thermal stability of the composites. This finding is in line with Kalam *et al.* [27] who observed a similar trend in TG for OPEFB fiber polypropylene composite. In addition, at similar fiber content bleached fiber composites display an early decomposition temperature compared to untreated fiber composites. Two remarks can be deduced from those results: the incorporation of OPEFB fiber reduced the decomposition temperature of the composites and bleaching treatment did not improve the overall thermal stability.

## 3. Experimental Section

### 3.1. Materials

Poly(lactic acid) pellets with density 1.24 g/cm^3^ (Grade 4060D) was supplied by Nature Work LLC (Minnetonka, MN, USA). The OPEFB fiber was obtained from Sabutek (M) Sdn. Bhd. (Perak, Malaysia). Hydrogen Peroxide (30%) was purchased from J.T. Baker (Center Valley, PA, USA) and sodium hydroxide from R&M Chemicals (Essex, UK). Masterbatch colorant (28 wt. % pigment) was supplied by Dongguan Liangsu Masterbatch Technology (Guangdong, China). All the materials were used as received.

### 3.2. Fiber Bleaching

The OPEFB fiber was soaked in distilled water at 70 °C for 4 h to remove excessive wax and other impurities. The fiber was dried in an oven at 60 °C for 24 h before being ground to a 300 µm fiber size. These dried fibers were designated as untreated fibers (UTF). The untreated OPEFB fiber was subjected to alkaline bleaching at 5.0 vol. % hydrogen peroxide concentration at pH 11 and at 70 °C for 90 min. NaOH was used to obtain the alkaline condition. Next, the fiber was washed with distilled water until neutral pH was obtained. After washing, the fibers were dried in an oven at 60 °C for 24 h. These fibers are labeled as bleached fibers (BF).

### 3.3. Preparation of Composites

Prior to the mixing process, PLA pellets and OPEFB fiber were dried at 60 °C for 24 h in an oven to remove moisture. The PLA/untreated fiber composites were prepared by using an internal mixer (Thermo Haake, Karlsruhe, Germany) at 160 °C for 15 min with a rotor speed of 50 rpm. The weight ratios of PLA/untreated fiber were 100/0, 90/10, 80/20, 70/30, 60/40 and 50/50. The biocomposites were then compress molded using a hydraulic hot press (Hsin-Chi Machinery Company Ltd., Hsinchu Hsien, Taiwan) at 160 °C with a 110 kg/cm^3^ pressure. The same procedure was repeated for the preparation of PLA/bleached fiber and PLA/bleached fiber/colorant (0.5 wt. %) composites. Subsequently, the composites underwent characterization for mechanical, thermal and morphological properties.

### 3.4. Characterization of Fiber and Composites

#### 3.4.1. SEM Analysis

Scanning electron microscopy (SEM JEOL model JSM-6300F, JEOL Ltd., Tokyo, Japan) with an acceleration voltage of 20 kV was used to assess the surface topography of fibers and tensile fracture surface of neat PLA and its composites. The specimens were coated with gold to make them electrically conductive.

#### 3.4.2. FTIR Analysis

The untreated and bleached fibers were analyzed using an FTIR Spectrometer (Perkin-Elmer: Model 1000 Series, Perkin Elmer, Waltham, MA, USA) instrument equipped with a universal attenuated total reflectance (UATR) accessory. The spectra were recorded between a range of wavenumbers from 4000 to 280 cm^−1^ with a 4 cm^−1^ spectral resolution.

#### 3.4.3. XRD Analysis

An X-ray diffractometer Shimadzu XRD 6000 (Tokyo, Japan) was employed to study the crystallinity for both treated and untreated fiber. The X-ray wavelength was 1.5405 Å and the diffraction patterns were recorded in step-scan mode (2θ range: 10°–40°) with a scanning speed of 2°/min. The calculation for crystalline index of cellulose (Cr*I*) for treated and untreated fiber was determined based on Segal empirical method [13] stated as follows (Equation (2)):

Cr*I* (%) = [(*I*_200_ − *I*_non-Cr_)/*I*_200_] × 100% (2)

where *I*_200_ is the peak intensity corresponding to cellulose and *I*_non-Cr_ represents the non-crystalline region.

#### 3.4.4. TGA

A TG Analyzer, Perkin Elmer TGA7 (Perkin Elmer, Waltham, MA, USA) was used to study the thermal stability of the neat PLA and its composites. TGA measures changes in the weight of the sample as a function of temperature and/or time. The samples were heated from 25 to 500 °C at constant heating rate of 10 °C/min under nitrogen atmosphere (flow rate: 50 mL/min). The weight loss of the sample was plotted as a function of temperature.

#### 3.4.5. Tensile Test

Neat PLA and PLA composite sheets were cut into dumbbell shaped specimens prior to use. The width and the thickness of the specimens were measured at three different points and an average value was calculated. The tensile properties: tensile strength, elongation at break and tensile modulus were determined using an Instron Universal Testing Machine (Model 4302 Series IX, Instron, Norwood, MA, USA) according to American Society for Testing and Materials (ASTM) D638 standard. Seven samples were tested for each reference.

#### 3.4.6. Izod Impact Test

Neat PLA and PLA composites were tested. Specimens with dimensions 63.50 mm × 12.70 mm × 3.00 mm were prepared. Impact test was performed using an izod impact tester (International Equipment, Mumbai, India) with a pendulum weighing 453 g (1.0 lb) based on ASTM D256 standard. Seven specimens were tested for each reference.

## 4. Conclusions

The effects of fiber bleaching treatment on the mechanical, thermal and morphological properties of OPEFB fiber/PLA composites have been investigated. Mechanical properties of the composites made with bleached fibers showed an improvement compared to untreated fiber. Bleaching of fiber is effective at removing surface impurities, lignin and hemicellulose, thereby producing brighter color and rougher fiber surface which promotes fiber/matrix adhesion as depicted by SEM micrographs. On the other hand, bleaching treatment did not improve thermal stability of the composites. Nevertheless, incorporation of bleached fiber into PLA resulted in a better appearance of the composites, and made further modification with colorants possible.
